# The contribution of fisheries and aquaculture to the global protein supply

**DOI:** 10.1007/s12571-021-01246-9

**Published:** 2022-01-20

**Authors:** Claude E. Boyd, Aaron A. McNevin, Robert P. Davis

**Affiliations:** 1 School of Fisheries, Aquaculture and Aquatic Sciences, Auburn University, Auburn, Alabama 36849 USA; 2 World Wildlife Fund, Washington, D.C USA

**Keywords:** Animal-source protein, Aquaculture production, Capture fisheries production, Global protein production, Resource use efficiency, Animal feeds

## Abstract

The contribution of aquatic animal protein to the global, animal-source protein supply and the relative importance of aquaculture to capture fisheries in supplying this protein is relevant in assessments and decisions related to the future of aquatic food production and its security. Meat of terrestrial animals, milk, and eggs resulted in 76,966 Kt crude protein compared with 13,950 Kt or 15.3% from aquatic animals in 2018.While aquaculture produced a greater tonnage of aquatic animals, capture fisheries resulted in 7,135 Kt crude protein while aquaculture yielded 6,815 Kt. Capture fisheries production has not increased in the past two decades, and aquaculture production must increase to assure the growing demand for fisheries products by a larger and more affluent population. We estimated based on *status quo* consumption, that aquaculture production would need to increase from 82,087 Kt in 2018 to 129,000 Kt by 2050 to meet the demand of the greater population. About two-thirds of finfish and crustacean production by aquaculture is feed-based, and feeds for these species include fishmeal and fish oil as ingredients. Aquaculture feeds require a major portion of the global supply of fishmeal and fish oil. An estimated 71.0% of fishmeal and 73.9% of fish oil are made from the catch with the rest coming from aquatic animal processing waste. The catch of small, pelagic fish from the ocean is not predicted to increase in the future. Aquaculture should reduce its fishmeal and oil use to lessen its dependency on small wild fish important to the integrity of marine food webs and food security for the poor in many coastal areas. Fishmeal and fish oil shortages for use in aquaculture feed will result in a limit on production in the future if goals to lessen their use in feeds are not met.

## Introduction

**Fig. 1:**
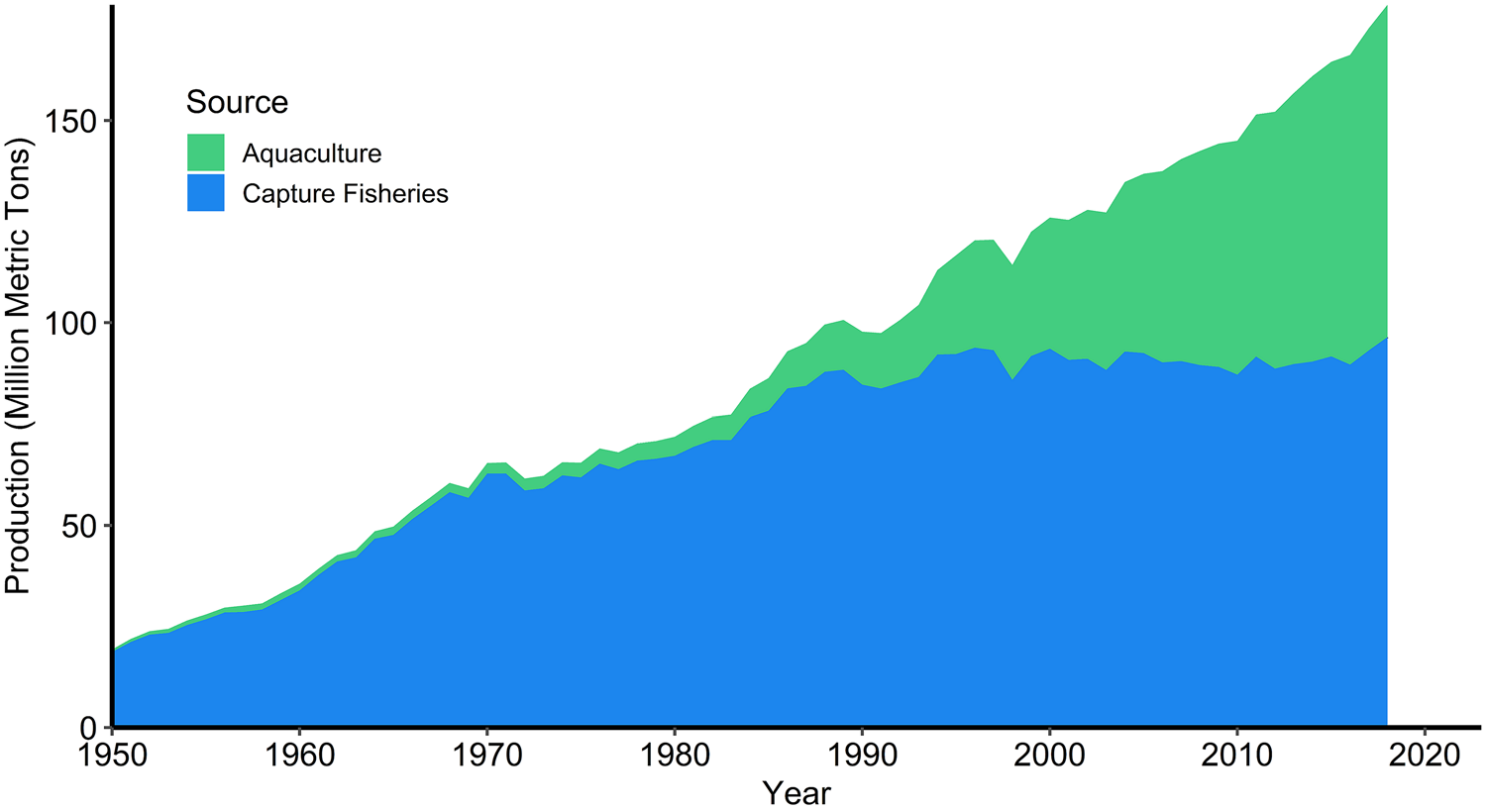
World production of capture fisheries and of aquaculture from 1950 to 2018 (source: FAO, 2020a)

Agriculture has provided humans with terrestrial, animal-source food for at least 5,000 years (Larsen, 2003; Modlinska & Pisula, 2018), but fish and other aquatic animals have traditionally been caught from the ocean and inland waters. Farming of aquatic animals has been done for more than 2,000 years, although this practice did not become a noticeable factor in global meat production until the twentieth century (Stickney, 2000). Global aquaculture production has increased rapidly since the 1950s while global capture fisheries production has shown no trend of increase after the early 1990s (Fig. 1). Aquaculture production surpassed capture fisheries production for human consumption in 2016 (FAO, 2020a), and it contributed 52% of the total harvest weight of aquatic animals for human consumption in 2018 (FAO, 2020a, c).

The amount of consumed meat from an animal is less than the animal’s weight because certain of its parts are not suitable for food or are not preferred for food (Boler & Woerner, 2017; Wright, 2016). The importance of differences in meat yields of animals is evident from a study by Edwards et al. (2019) in which they found that although aquaculture production of animals for human consumption exceeds capture fisheries production, more meat was produced by the capture fisheries as a result of differences in meat yield among different species within the two sectors.

Meat is important in human diets because a dietary portion of it typically has a higher protein concentration with a better balance of essential amino acids than does an equal size portion of a plant-source food (Wyness, 2016, McNeill et al., 2017; Klurfeld, 2018; Tilami & Samples, 2018). Milk and eggs have lower protein concentrations than do meats, but they have an excellent balance of essential amino acids (Muehlhoff et al., 2013; Nassar, 2016). The supply of protein available to humans is a critical factor in global food security (Henchion et al., 2017), and information about the contribution of aquatic animals to food supply is relevant to a better understanding of this aspect of the world food system.

According to FAO (2020c), aquaculture and fisheries combined accounted for 17% of total animal-source protein for human consumption. The FAO statement did not separate the protein contribution of capture fisheries and aquaculture to the supply. It is important to know the contribution of each of the two sources of aquatic animal protein, and the amounts of protein from each of the major animal groups, i.e., fish, crustaceans, and molluscs, of capture fisheries and of aquaculture.

Oceans are overfished (FAO, 2020c; Sumaila & Tai, 2020), but there are those (such as Duarte et al., 2009; Costello et al., 2020) who propose that mariculture (ocean aquaculture) in combination with adequate ocean conservation measures would allow the ocean to provide more protein for humans in the future. There is scant prospect for increasing the capture from the ocean while applying the current methods of fishing (Costello et al., 2020).

The changing climatic conditions are expected to worsen in years to come, and this challenge provides opportunity to create new systems of food production and to abandon or lessen the use of some systems in efforts to conserve land, water, and energy as well as to reduce greenhouse gas emissions (GHGs) or increase carbon sequestration (Campbell et al., 2016; Gephart et al., 2021; Rosenzweig et al., 2021; Thiault et al., 2019). This is important because food production uses 38% of land (FAO, 2016) and 70% of freshwater (FAO, 2016), while resulting in 25–35% of global greenhouse gas emission (IPCC, 2013; Tubiello et al., 2014).

Capture fisheries and aquaculture are rather minor components of global use of most resources. Boyd and McNevin (2015) estimated land use by aquaculture as 0.17% of the global land area, and consumptive water use as 0.82% of renewable, available freshwater. Consumptive water use does not include the “so-called” green water (rainwater) that falls on agricultural fields and aquaculture ponds only to evaporate. Verdegem and Bosma (2009) included green water and concluded that 3.2% of global freshwater use was by aquaculture. Boyd and McNevin (2015) estimated that capture fisheries and aquaculture produced 0.61% and 0.49% of global GHG emissions, respectively. A more recent estimate by Macleod et al. (2020) also gave an estimate of GHG emissions by aquaculture as 0.49% of global emissions, but they did not give an estimate for capture fisheries. However, aquaculture dominates the global consumption of fishmeal and fish oil made from small, oceanic fish. Aquaculture production needed 18.3% of fish captured from the ocean in 2017 for feed ingredients (Naylor et al., 2021). Most of the fish used to make ingredients for aquaculture feed are suitable for human food (Cashion et al., 2017), and Taylor et al. (2019) noted that in some coastal areas, declining ocean fisheries are threatening the food security of coastal residents.

The COVID-19 pandemic has demonstrated the fragility of the global food system and food companies are balancing on the razor’s edge between function and collapse. Sarkis et al. (2020) noted that there is now a window for transitioning to sustainable supply chains in the aftermath of COVID-19 that includes rethinking vulnerabilities created by over-reliance on ‘just-in-time’ or ‘business-as-usual’ practices. Some businesses are preparing for the next shock to the system by buffering company operations, because greater resilience in food systems are needed to support food security against unexpected events (Kahiluoto, 2020; Savary et al., 2020).

Consumers need to be informed about the environmental sustainability of different foods in order to make informed decisions when purchasing. Impulsive consumers can be influenced by media reports on why one protein should be abandoned for another and lose the context of the trade-offs among resource use and environment impacts which occur when shifting from one food to another. A consistent way to value these trade-offs needs to be demonstrated, but it is challenging to achieve this because of the different units of measure applied in reporting resource use, i.e., land (area/t of production), water (m^3^/t), and energy (GJ/t). The comparative ecological benefit of savings per unit of land, water, and energy is needed for comparing resource use tradeoffs. Such decisions should be based on valuation of the comparative importance of the resources in the food system and to environment sustainability (Boyd & McNevin, 2015).

As part of the effort to make food production more efficient and sustainable, information on the annual amounts of animal-source protein from all sources are needed. But in particular, details on the amounts of protein from the different groups of aquatic animals captured from the ocean and produced by aquaculture are lacking. The present study was conducted to determine the amounts of human-consumed protein produced in 2018 by different species groups within the capture fisheries and the aquaculture sectors. These data were compared with similar determinations of animal-source protein production from traditional meat animals, milk, and eggs. These comparisons should be useful in future efforts to decide upon strategies for increasing the role of aquaculture in meeting the future demand for protein to supply the ever-growing human population (Béné et al., 2015; De Silva, 2016; Irwin et al., 2021; Mahfuzul & Dey, 2017).

## Methodology

### Definition of terminology

Definitions of some basic terms used throughout this paper may be helpful. Capture fisheries include finfish, crustaceans, molluscs, other kinds of animals, and seaweeds taken from the ocean, estuaries, or inland waters by fishing techniques or other methods. Aquaculture refers to finfish, crustaceans, molluscs, other kinds of animals, and seaweeds produced by farming techniques. Finfish are reared in ponds, cages, raceways, tanks or other means of confinement. Mollusc spat can be placed on ropes, various types of underwater structures, or laid on shallow bottom areas for grow-out. Seaweed propagules are attached to ropes to provide mooring. The terms aquatic animals, aquatic plants, and aquatic meats also are sometimes used to refer collectively to all products of the capture fisheries, of aquaculture, or both combined. The amount of the fish from capture fisheries used to make fishmeal and fish oil is called the reduction fishery, but is sometimes called the feed fish fishery.

### Background data used in crude protein calculations

#### Animal-sources for human consumption

Estimates in thousands of tonnes (Kt) of terrestrial meat production reported in carcass weights and amounts of milk and eggs produced (Tables 1 and 2) were taken from the FAO agricultural database (FAO, 2020b). The harvest weights (Kt) in 2018 of specific species or species groups of aquatic animals from the capture fisheries and aquaculture were obtained from the FAO fisheries and aquaculture database (FAO, 2020a) and the *Status of World Fisheries and Aquaculture* biannual report (FAO, 2020c) are summarized in Table 3. While FAO separates production for both capture fisheries and aquaculture into marine, brackish water, and inland categories, it better fitted the purposes here to rearrange the data into only capture fisheries and aquaculture production (Table 3). The capture fishery in 2018 included 2,221 species, but 40 species or species groups gave about 80% of total sector production. By contrast, 600 aquaculture species were recorded, but 12 species or species groups provided 87% of total production by aquaculture (FAO, 2020a).

**Table 1:** Global production of terrestrial meat animals, meat portion, crude protein concentration in meat, and yield of crude protein

			Crude protein	
Category	Carcass weight^1^ (Kt)	Meat portion^2^ (%)	In meat portion^3^ (%)	Yield (Kt)
Chicken	118,017	70.0	18.6	15,366
Pig	110,110	65.0	13.9	9,948
Cattle	68,314	57.5	17.3	6,796
Sheep	9,922	72.5	20.0	1,439
Goats	6,253	63.8	20.5	818
Turkey	5,992	72.6	19.0	826
Ducks	4,858	67.4	19.0	622
Buffalo	4,290	69.5	20.3	605
Geese and guinea	2,761	67.7	19.0	355
Game	2,049	61.5	22.9	289
Horse, mule, and ass	933	74.5	21.2	147
Rabbit	884	55.9	20.3	100
Camel and camelids	686	56.0	19.6	75
Bird	19	57.8	23.7	3
Rodents	19	53.8	23.8	2
Total crude protein				37,391

^1^FAO (2020b)

^2^Chicken—Hayse and Marion (1973); pig, cattle, sheep—Raines (2017); goats—Webb (2014), Schoenian (2020); turkey—Miller (1965); ducks—Kokosyński et al. (2020); buffalo—Peixoto et al. (2012); geese and guinea—Gumulka and Poltowicz (2020); game—Kay et al. (1981); horse, mule, ass—de Paulo et al. (2014); rabbit—Ghosh and Mandal (2008); camel and camelids—Yousif and Babiker (1989); bird—Kokoszyński et al. (2020); rodents—de Figueiredo et al. (2020)

^3^Chicken, pigs, cattle, sheep, goats, turkey, ducks, geese, guinea—USDA (2021a); buffalo—Navenna and Kiran (2014); game—Kay et al. (1981); horse, mule, ass—de Palo et al. (2014); rabbit—Ghosh and Mandal (2008); camel and camelids—Kadim et al. (2014); bird—Kokosyński et al. (2020); rodents—de Figueiredo et al. (2020)

**Table 2:** Global production of eggs and milk, consumable portion, crude protein concentration in consumable portion and yields of crude protein

			Crude protein	
Category	Production^1^ (Kt)	Consumable portion^2^ (%)	In consumable portion^3^ (%)	Yield (Kt)
Egg				
Hen	83,484	90.3	10.8	8,142
Other	6,040	90.9	9.9	544
Total egg protein				8,686
Milk				
Cow	715,923	100.0	3.4	24,341
Buffalo	133,752	100.0	3.9	5,216
Goat	19,910	100.0	3.3	657
Sheep	10,587	100.0	5.4	572
Camel	3,111	100.0	3.3	103
Total milk protein				30,889

^1^FAO (2020b)

^2^Egg—Sun et al. (2019)

^3^Egg—Sun et al. (2019); cow milk—Franzoi et al. (2019); buffalo milk—Mohamed et al. (2011); sheep and goat milk—Ferro et al. (2017); camel milk—Bouhaddaoui et al. (2019)

**Table 3:** World fisheries and aquaculture production in 2018 in kilotonnes of harvested weight (source: FAO, 2020c)

Category	Capture	Aquaculture	Total
Aquatic animals			
Finfish	61,826^1^	54,279	116,105
Finfish for non-food uses	22,100^2^	–-	22,100
Crustaceans	5,979	9,387	15,366
Mollusc	5,959	17,511	23,470
Others	531	910	1,441
Total animals	96,395	82,087	178,482
Total animals for human food	74,295	82,087	156,382
Seaweeds	906	31,480	32,386
Total production	97,301	113,567	210,868

^1^Includes about 12,000 Kt of freshwater finfish used almost entirely for human food

^2^The largest portion (17,700 Kt) was reduced to fishmeal and fish oil. The remainder was for ornamental fish, bait, fry and fingerlings for grow-out in aquaculture, pet food, live feed for aquaculture, and a few other uses (FAO, 2020c)

For the purpose of estimating crude protein amounts, capture fisheries production, because of its larger number of species was condensed into seven categories (Table 4), while aquaculture production was divided into 15 categories with 11 being major species or species groups (Table 5). All types of molluscs were considered a single species group, because scant information on meat yields and protein concentrations for individual species was found.

**Table 4:** Global production of aquatic animals by capture fisheries, meat portion, crude protein in meat, and crude protein yields

	Harvested	Meat	Crude protein	
Category	amount^1^ (Kt)	portion^2^ (%)	In meat portion^3^ (%)	Yield (Kt)
Marine finfish	49,826	55.1	19.4	5,326
Crustaceans				
Shrimp	3,200	52.8	19.8	345
Other	2,797	21.0	18.2	107
Mollusc	5,959	16.2	11.8	114
Other	531	41.8	17.9	40
Freshwater finfish	12,000	54.5	18.4	1,203
Total crude protein				7,135

^1^FAO (2020c)

^2^FAO (1983); Carpo et al. (2004)

^3^FAO (1983); Musaiger and Al-Rumaidh (2005); Fernandez et al. (2018); Celik et al. (2011); Venugopal and Gopakumar (2017); https://www.fishchoice.com/buying-guide/snow-crab

**Table 5:** Global production of aquatic animals by aquaculture, meat portion, crude protein in meat, and crude protein yield

			Crude protein	
Category	Amount^1^ (Kt)	Edible portion^2^ (%)	In edible portion^3^ (%)	Amount (Kt)
Finfish				
Carp	28,866	56.2	20.1	3,261
Tilapia	6,031	34.2	19.8	408
Catfish	5,781	47.1	17.7	482
Atlantic salmon	2,436	60.0	20.6	301
Milkfish	1,327	46.7	17.8	110
Rainbow trout	848	64.0	20.5	111
Other	8,990	51.3	19.7	909
Mollusc	17,511	16.0	11.4	319
Crustaceans				
White-leg shrimp	4,966	52.4	21.3	554
Red swamp crayfish	1,711	20.7	19.1	68
Mitten crab	757	23.5	18.4	33
Black tiger shrimp	751	55.0	17.5	72
Freshwater shrimp	472	45.0	18.8	40
Other	730	39.3	18.9	54
Other animals	919	51.3	19.7	93
Total crude protein				6,815

^1^FAO (2020a)

^2^Carp—Mahboob et al. (2004), Raghunath et al. (2016), Sahu et al. (2013), Geri et al. (1995); tilapia—Sahu et al. (2017), Paul et al. (2018), Khalil et al. (1980); catfish—Argue et al. (2003), Wu and Lillard (1998), Men et al. (2005), Hoffman et al. (1993); Atlantic salmon—MΩWI (2019); milkfish—Lingam et al. (2019); rainbow trout—Lanari and D’agaro (2002), Krause et al. (2002); mollusc—Venugopal and Gopakumar (2017); white-leg shrimp—Kim et al. (2011); red swamp crayfish—Mona et al. (2000); Hamdi and El-Monem (2006); mitten crab—Shao et al. (2014); black tiger shrimp—Fernandez et al. (2018); freshwater shrimp—Hung and Nguyen (2014)

The typical yields of meat for consumption by humans from processing carcasses of terrestrial animals and whole aquatic animals, and crude protein concentrations for the different protein sources also are presented in Tables 1, 2, 4, and 5. The crude protein concentrations reported by the literature sources (Tables 1, 2, 4 and 5) were made by either the Kjedahl method or the Dumas method, which give similar results (Simonne et al., 1997; Müller, 2017).

#### Fishmeal, fish oil and seaweeds

The term reduction fishery is often used to describe the part of capture fisheries reduced to the co-products fishmeal and fish oil. The average recoveries from reduction are 20.8% for fishmeal and 4.4% for fish oil (IFFO, 2017). Fishmeal made from the reduction fishery contains 60% to 72% crude protein with 65% being a typical concentration (Cho & Kim, 2010). Fish oil does not contain protein (USDA, 2021b).

An estimated 5,600 Kt fishmeal were produced in 2018 resulting in 1,600 Kt of fish oil as a co-product (EUMOFA, 2021). Fishmeal and fish oil also are made by rendering trimmings, bycatch, and other aquatic animal processing waste comprised about 29.0% of meal and 26.1% of oil production in 2016 (IFFO, 2020). An estimated 65.8% of processing wastes for this came from the capture fisheries and 34.2% from aquaculture, but the amounts from inland and marine sources were not estimated (IFFO, 2020). Recovery rates for fishmeal and fish oil from processing waste are 26.5% and 4.2%, respectively, and similar to those for whole fish as calculated from data in Jackson and Newton (2016). However, fishmeal and fish oil from rendering processing waste is of lower protein concentration than that made from whole fish, because the waste is high in ash (inorganic) content from bone (Coppola et al., 2021; Ghaly et al., 2013). For example, the yield of fishmeal from processing waste was 35.8% for African catfish (Likitrattanaporn, 2016), 54.8% from tilapia (Dale et al., 2004), 61.9% from cod and saithe (Ween et al., 2017), and 40–60% for unspecified species of fish (Krishnamoorthy, 2018). The average of these estimates was 50.5% crude protein.

#### Seaweeds

Seaweeds also are sources of protein from the ocean and 97% of the harvest weight is from aquaculture (Table 3). Seaweeds have an average dry matter concentration of 17% (Rasyid, 2017; Wickham et al., 2019), and the dry matter has an average crude protein concentration of 16.7% (Angell et al., 2015; Biancarosa et al., 2016; Rasyid, 2017).

### Calculations of crude protein

Equations were made for calculating the total amount of crude protein, also in thousands of tonnes (Kt), from individual sources using the background data from 2.1.1–2.1.3 above, and are provided below.

1 $$\mathrm{Terrestrial}\;\mathrm{meat}\;\mathrm{animals},\quad{\mathrm{CP}}_{\mathrm i}=\left({\mathrm W}_{\mathrm{ci}}\right)\left({\mathrm Y}_{\mathrm{mi}}/100\right)\left({\mathrm{cp}}_{\mathrm{mi}}/100\right)$$

$$\mathrm{where}\;{\mathrm{CP}}_{\mathrm i}=\mathrm{crude}\;\mathrm{protein}\;\mathrm{from}\;\mathrm{animal}\;\mathrm i\;(\mathrm{Kt}),$$

$${\mathrm W}_{\mathrm{ci}}=\mathrm{carcass}\;\mathrm{production}\;\mathrm{of}\;\mathrm{animal}\;\mathrm i\;(\mathrm{Kt}),$$

$${\mathrm Y}_{\mathrm{mi}}=\mathrm{edible}\;\mathrm{meat}\;\mathrm{yield}\;\mathrm{from}\;\mathrm{carcass}\;\mathrm{of}\;\mathrm{animal}\;\mathrm i\;(\%),$$

$$\begin{aligned}{\mathrm{cp}}_{\mathrm{mi}}= &\; \mathrm{crude}\;\mathrm{protein}\;\mathrm{concentration}\;\mathrm{in}\;\mathrm{edible}\;\mathrm{meat}\;\mathrm{of}\;\mathrm{animal}\;\mathrm i\;(\%).\end{aligned}$$

2 $$\mathrm{Aquatic}\;\mathrm{animals},\quad {\mathrm{CP}}_{\mathrm i}=\left({\mathrm W}_{\mathrm i}\right)\left({\mathrm Y}_{\mathrm{mi}}/100\right)\left({\mathrm{cp}}_{\mathrm{mi}}/100\right)$$

$$\mathrm{where}\;{\mathrm W}_{\mathrm i}=\mathrm{live}\;\mathrm{weight}\;\mathrm{production}\;\mathrm{of}\;\mathrm{animal}\;\mathrm i\;(\mathrm{Kt}),$$

$${\mathrm Y}_{\mathrm{mi}}=\mathrm{edible}\;\mathrm{meat}\;\mathrm{yield}\;\mathrm{from}\;\mathrm{whole}\;\mathrm{animal}\;\mathrm i\;(\%).$$

3 $$\mathrm{Eggs},\quad{\mathrm{CP}}_{\mathrm{ei}}=\left({\mathrm W}_{\mathrm{ei}}\right)\left({\mathrm Y}_{\mathrm{ywi}}/100\right)\left({\mathrm{cp}}_{\mathrm{ywi}}/100\right)$$

$$\mathrm{where}\;{\mathrm{CP}}_{\mathrm{ei}}=\mathrm{crude}\;\mathrm{protein}\;\mathrm{in}\;\mathrm{eggs}\;\mathrm{for}\;\mathrm{animal}\;\mathrm i\;(\mathrm{Kt}),$$

$${\mathrm W}_{\mathrm{ei}}=\mathrm{weight}\;\mathrm{of}\;\mathrm{eggs}\;\mathrm{from}\;\mathrm{animal}\;\mathrm i\;(\mathrm{Kt}),$$

$${\mathrm Y}_{\mathrm{ywi}}=\mathrm{yolk}\;\mathrm{and}\;\mathrm{white}\;\mathrm{for}\;\mathrm{eggs}\;\mathrm{of}\;\mathrm{animal}\;\mathrm i\;(\%),$$

$$\begin{aligned}{\mathrm{cp}}_{\mathrm{ywi}}= &\; \mathrm{crude}\;\mathrm{protein}\;\mathrm{concentration}\;\mathrm{in}\;\mathrm{yolk}\;\mathrm{and}\;\mathrm{white}\;\mathrm{of}\; \\ & \mathrm{eggs}\;\mathrm{of}\;\mathrm{animal}\;\mathrm i\;(\%).\end{aligned}$$

4 $$\mathrm{Milk},\quad {\mathrm{CP}}_{\mathrm{mi}}=\left({\mathrm{W}}_{\mathrm{mi}}\right)\left({\mathrm{cp}}_{\mathrm{mi}}/100\right)$$

$$\mathrm{where}\;{\mathrm{CP}}_{\mathrm{mi}}=\mathrm{crude}\;\mathrm{protein}\;\mathrm{in}\;\mathrm{milk}\;\mathrm{of}\;\mathrm{animal}\;\mathrm i\;(\mathrm{Kt}),$$

$${\mathrm W}_{\mathrm{mi}}=\mathrm{weight}\;\mathrm{of}\;\mathrm{milk}\;\mathrm{from}\;\mathrm{animal}\;\mathrm i\;(\mathrm{Kt}),$$

$${\mathrm{cp}}_{\mathrm{ywi}}=\mathrm{crude}\;\mathrm{protein}\;\mathrm{concentration}\;\mathrm{in}\;\mathrm{milk}\;\mathrm{of}\;\mathrm{animal}\;\mathrm i\;(\%).$$

Amounts of fishmeal and crude protein from fishmeal were calculated as:

5 $${\mathrm{FM}}_{\mathrm{rf}}= \frac{{\mathrm{P}}_{\mathrm{rf}}}{4.8}{\mathrm\quad{\mathrm {and}}\quad {\mathrm {FM}}}_{\mathrm{pw}}= \frac{{\mathrm{P}}_{\mathrm{pw}}}{4.8}$$

$$\begin{aligned}\mathrm{where}\;{\mathrm{FM}}_{\mathrm{rf}}\;\mathrm{and}\;{\mathrm{FM}}_{\mathrm{pw}}=&\;\mathrm{fish}\;\mathrm{meal}\;\mathrm{from}\;\mathrm{reduction}\;\\&\mathrm{fishery}\;\mathrm{and}\;\mathrm{from}\;\mathrm{processing}\;\mathrm{waste},\\ &\mathrm{respectively}\;(\mathrm{Kt}),\end{aligned}$$

$$\begin{aligned}{\mathrm P}_{\mathrm{rf}}\;\mathrm{and}\;{\mathrm P}_{\mathrm{pw}}=&\;\mathrm{amounts}\;\mathrm{from}\;\mathrm{reduction}\;\mathrm{fishery}\;\mathrm{and}\;\mathrm{from}\;\\&\mathrm{processing}\;\mathrm{waste}, \mathrm{respectively}\;(\mathrm{Kt}),\end{aligned}$$

$$4.8=\mathrm{wild}\;\mathrm{fish}/\mathrm{fishmeal}\; \mathrm {ratio}.$$

6 $$\mathrm{CP_{rf}=(FM_{rf})(cp_{rf}/100) \quad or \quad CP_{pw}=(FM_{pw})(cp_{pw}/100)}$$

$$\begin{aligned}&\mathrm{where}\;{\mathrm{CP}}_{\mathrm{rf}}\;\mathrm{and}\;{\mathrm{CP}}_{\mathrm{pw}}\;\mathrm{are}\;\mathrm{amounts}\;\mathrm{of}\;\mathrm{crude}\;\mathrm{protein}\;\mathrm{from}\;\\ &\mathrm{reduction}\;\mathrm{fishery}\;\mathrm{and}\;\mathrm{from}\;\mathrm{processsing}\;\mathrm{waste}\;(\mathrm{Kt}),\end{aligned}$$

$$\begin{aligned}{\mathrm{cp}}_{\mathrm{rf}}\;\mathrm{and}\;{\mathrm{cp}}_{\mathrm{pw}}=&\;\mathrm{protein}\;\mathrm{concentrations}\;\mathrm{in}\;\mathrm{fishmeal}\;\mathrm{from}\;\\&\mathrm{reduction}\;\mathrm{fishery}\;\mathrm{and}\;\mathrm{in}\;\\&\mathrm{processing}\;\mathrm{waste},\;\mathrm{respectively}\;(\%).\end{aligned}$$

Fish oil production was estimated as:

7 $${\mathrm{FO}}_{\mathrm{rf}}=\frac{{\mathrm{P}}_{\mathrm{rf}}}{22.5}\quad \mathrm{or}\quad \mathrm{FO}_{\mathrm{pw}}= \frac{{\mathrm{P}}_{\mathrm{w}}}{23.7}$$

$$\mathrm{where}\;{\mathrm{FO}}_{\mathrm{rf}}=\mathrm{amount}\;\mathrm{of}\;\mathrm{fish}\;\mathrm{oil}\;\mathrm{from}\;\mathrm{reduction}\;\mathrm{fishery}\;(\mathrm{Kt}),$$

$${\mathrm{FO}}_{\mathrm{pw}}=\mathrm{amount}\;\mathrm{of}\;\mathrm{fish}\;\mathrm{oil}\;\mathrm{from}\;\mathrm{processing}\;\mathrm{waste}\;(\mathrm{Kt}),$$

$$22.5=\mathrm{wild}\;\mathrm{fish}/\mathrm{fish}\;\mathrm{oil}\;\mathrm{ratio},$$

$$23.7=\mathrm{processing waste}/\mathrm{fish oil ratio}.$$

The seaweed contribution of crude protein was:

8 $${\mathrm{CP}}_{\mathrm{s}}=\left({\mathrm{P}}_{\mathrm{s}}\right)\left({\mathrm{DM}}_{\mathrm{s}}/100\right)\left({\mathrm{cp}}_{\mathrm{s}}/100\right)$$

$$\mathrm{where}\;{\mathrm{CP}}_{\mathrm s}=\mathrm{amount}\;\mathrm{of}\;\mathrm{crude}\;\mathrm{protein}\;\mathrm{from}\;\mathrm{seaweed}\;\left(\mathrm{Kt}/\mathrm{yr}\right),$$

$${\mathrm{DM}}_{\mathrm s}=\mathrm{dry}\;\mathrm{matter}\;\mathrm{concentration}\;\mathrm{in}\;\mathrm{seaweed}\;\left(\%\right),$$

$${\mathrm{cp}}_{\mathrm s}=\mathrm{crude}\;\mathrm{protein}\;\mathrm{concentration}\;\mathrm{in}\;\mathrm{seaweed}\;\left(\%\right).$$

### Statistical analyses

A log–log scale linear regression was conducted for total carcass weights and harvest weights (x variables) and estimated amounts of crude protein. An ANCOVA type model with protein sources as a covariate (terrestrial, fisheries, and aquaculture) was not utilized after the interaction was found to be non-significant. A simple linear regression was used to model this relationship. A treemap plot (Kong et al., 2010) was used to visualize the distribution between the different sources of animal-based proteins in this study. Additionally, a spider or radar chart was used to examine the patterns in amino acid distributions in different sources of proteins representative of categories presented here. Spider charts are useful visualizations for seeing complex patterns in multiple, related variables (Wohlwend, 2012). All graphics and data analyses were conducted in R version 4.0.3 (R Core Team, 2020).

## Results

### Crude protein for human consumption

There were differences among amounts of carcass weights of the various terrestrial animals and the harvest weights of different aquatic animals. However, chickens, pigs, and beef cattle dominated terrestrial animal production (Table 1). The total harvest weight production of aquatic animals for human consumption of 156,382 Kt (Table 3) was much less than the total carcass weight of terrestrial animals of 330,107 Kt (Table 1). The combined global production of milk and eggs was 972,007 Kt (Table 2). Production of animals intended for human consumption was 82,087 Kt from aquaculture and 74,295 Kt from the capture fisheries (Table 3).

The animals differed in percentage meat yields and percentages of crude protein in their meat (Tables 1, 2, 4 and 5). These differences influenced the calculated yields of crude protein from carcass weights and harvest weights. For example, pigs were the largest category of carcass weight among terrestrial animals, but chicken meat provided more protein than did pig meat. This occurred mainly because, when all consumable meat cuts are considered, pig meat has a high fat content resulting in a lower overall percentage of crude protein which is located primarily in muscle tissue of meat cuts (Lonergan et al., 2019).

**Fig. 2:**
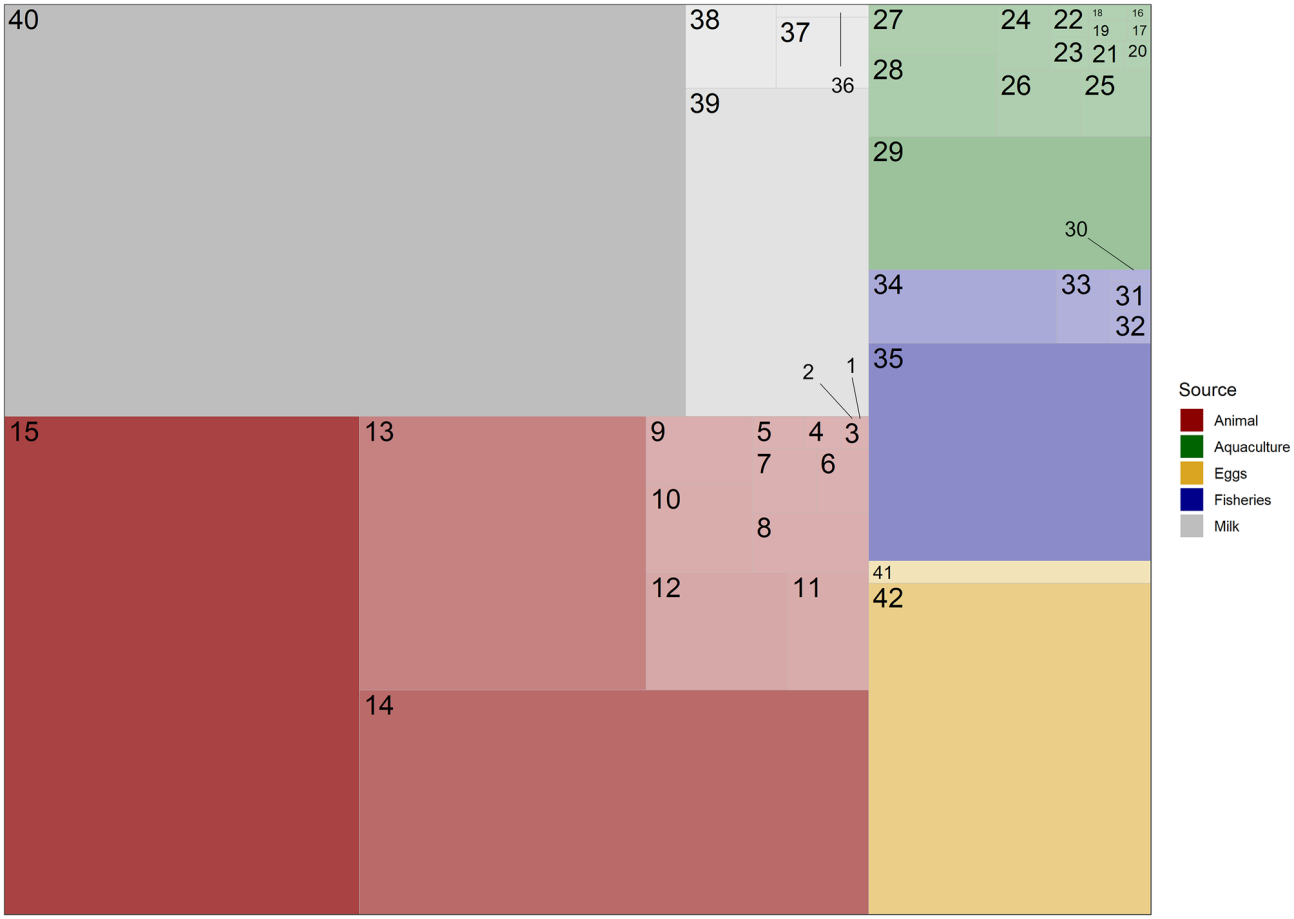
A treemap diagram of the contributions of different sources of protein to the overall global protein supply. Legend —1-Rodents, 2-Birds-other, 3-Camel and camelids, 4-Rabbit, 5-Horse, mule, and ass, 6-Game, 7-Geese and guinea, 8-Buffalo, 9-Ducks, 10-Goats, 11-Turkey, 12-Sheep, 13-Cattle, 14-Pork, 15-Chicken, 16-Red swamp crayfish, 17-Black tiger shrimp, 18-Freshwater shrimp, 19-Whiteleg shrimp, 20-Mitten crab, 21-Aquaculture–other, 22-Milkfish, 23-Rainbow trout, 24-Atlantic salmon, 25-Tilapia, 26-Catfish, 27-Molluscs–aquaculture, 28-Finfish, 29-Carps, 30-Fisheries–other, 31-Crustaceans excluding shrimp, 32-Molluscs–fisheries, 33-Shrimp–fisheries, 34-Freshwater finfish, 35-Marine finfish, 36-Camel milk, 37-Sheep milk, 38-Goat milk, 39-Buffalo milk, 40-Cow milk, 41-Eggs–other, 42-Chicken eggs

**Fig. 3:**
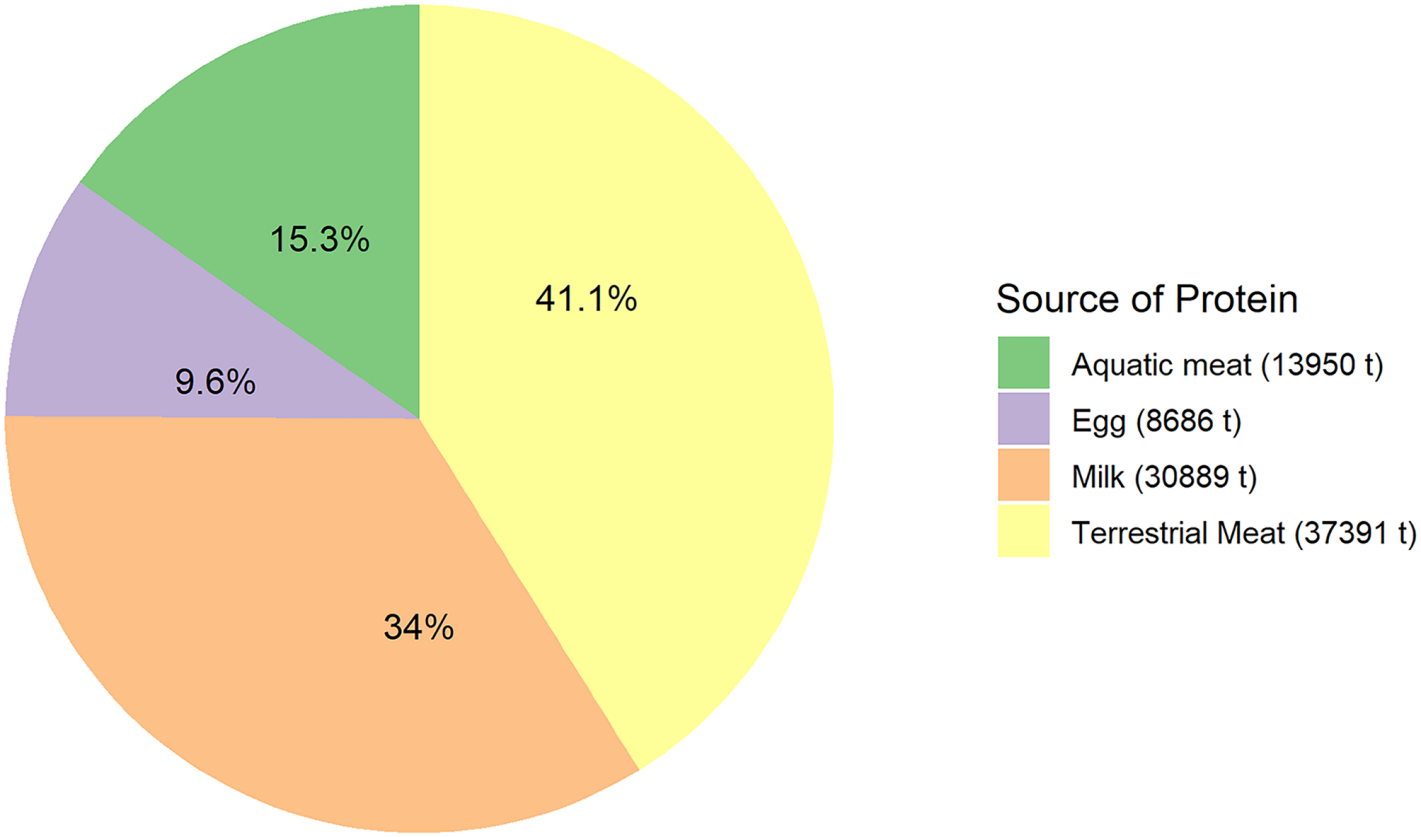
Global production of animal protein by the main sources

Amounts of crude protein originating from the different animal sources are listed in Tables 1, 2, 4 and 5; their sum was 90,916 Kt. The rectangular treemap diagram (Fig. 2) allowed a visual comparison of the amounts of protein from individual animal sources simultaneously, while Fig. 3 shows the sector contributions which were in the order: terrestrial meats > milk > eggs > capture fisheries > aquaculture. Each sector tended to be dominated by one or few categories, which suggests an overall lack of diversity in food production systems. For example, milk is almost entirely comprised of cow’s milk (> 80%), while eggs are dominated by chicken eggs (~ 94%). Terrestrial animal protein production is dominated by three animal groups, chicken, pigs, and cattle, which accounted for ~ 86% of this category. Capture fisheries was dominated by finfish (marine and freshwater), which together accounted for ~ 92% of capture fisheries production. However, the ten major species groups made up only about one-half of the finfish for human consumption (FAO, 2020c). There are more than 2,000 species, as previously pointed out, resulting in considerable species diversity of the production. Aquaculture protein production was dominated by a few species, such as carps, but to a lesser degree when compared to terrestrial animal agriculture.

Capture fisheries provided 7,135 Kt of crude protein compared with 6,815 Kt for aquaculture. The difference is small, about 4.6% less by aquaculture, and the contributions are equal for practical purposes. The reason that aquaculture, with 10.5% greater production, resulted in slightly less crude protein than did the capture fisheries was that only 8.0% of capture fisheries production for human consumption was from molluscs as compared to 21.3% for aquaculture. Molluscs have an average meat yield lower than that of most other aquatic meat animals, and a lower protein concentration (Tables 4 and 5). This results because molluscs have a low meat/shell ratio and the fresh meat has a high water content which dilutes its crude protein concentration (Moniruzzaman et al., 2021).

**Fig. 4:**
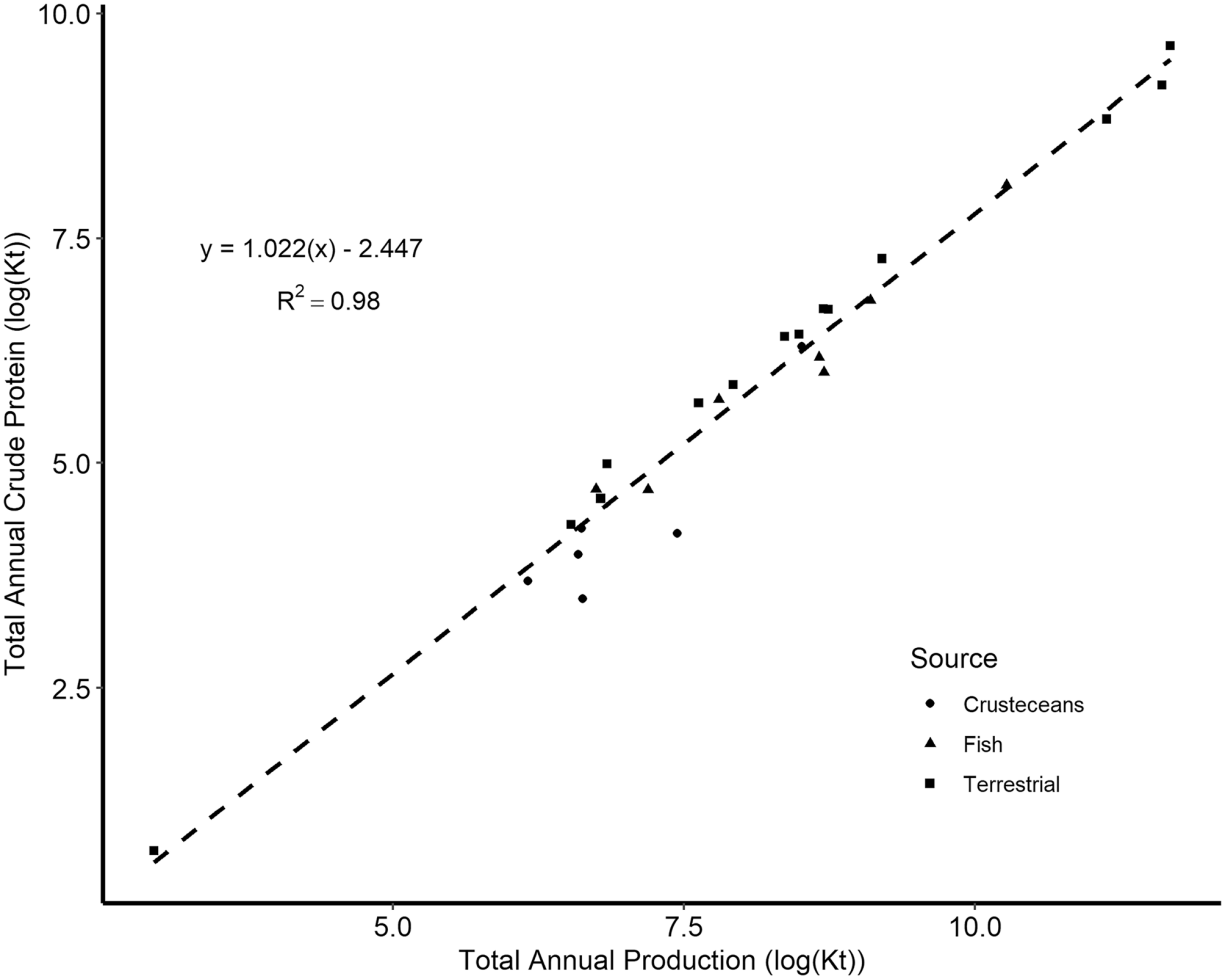
Relationship between carcass weights of terrestrial animals, harvest weights of aquatic animals, and crude protein production

Crude protein amounts were closely related to the quantities of carcass weights of terrestrial animals and the harvest weights of aquatic animals regardless of the two methods of reporting animal production (Fig. 4). The interaction between the protein source (fish, crustaceans, and terrestrial animals) and the slopes of the individual regression lines were not significant. This means the overall relationship shown in Fig. 4 is relatively the same regardless of the meat animal source of the protein. The equation in Fig. 4 could be used to obtain future estimates of global or regional protein from different animals.

### Crude protein for non-human food uses

According to FAO (2020c), about 22,100 Kt or 23.9% of the total capture fishery in 2018 were not used for human food (Table 3). The reduction fishery used for fishmeal and oil production consisted of an estimated 17,700 Kt of small fish which resulted in 3,688 Kt of fishmeal and 787 Kt of fish oil. The fishmeal contained 2,382 Kt crude protein. The remainder of the fish not intended for either human consumption or reduction was used as ornamental fish, bait, fry and fingerlings for grow-out in aquaculture, and for pet food (FAO, 2020c).

Fishmeal from the rendering of fish processing waste was estimated to be 1,912 Kt or 34.1% of the 2018 production. Fish oil production from rendering waste was 513 Kt or 39.4% of the 2018 production. The fishmeal from rendering contained an estimated 966 Kt crude protein, which was 28.9% of the crude protein in fishmeal produced in 2018.

### Crude protein from seaweeds

Crude protein from seaweeds was calculated to be 920 Kt of which 97.2% was from aquacultured seaweed. Around 80% of seaweed production is eaten directly as human food or processed to provide hydrocolloids such as carrageenan, agar, and alginates used as food and cattle feed additives. The remainder of the seaweed is used in diet pills, cosmetics, various industrial applications, and fertilizers (World Bank Group, n.d.; Ferdouse et al., 2018; West et al., 2016). There was not enough detail about seaweed use in human foods to estimate how much of the crude protein from seaweeds was consumed in human diets.

## Discussion

### Data quality

The reliability of the FAO fisheries and aquaculture database, and especially the data for China, has been questioned (Pauly & Froese, 2010; Pauly & Zeller, 2016, 2017). FAO has responded that while both under-reporting and over-reporting by some countries has occurred, the database is continually curated, updated, and revised. Moreover, FAO has an ongoing effort to improve country-level reporting (Ye et al., 2017). The authors have noted differences in a few data between subsequent editions of the FishStat J software (FAO, 2020a) which indicate revision, and we also suspect that the Chinese data have been consistently over-reported.

The FAO database contains annual fisheries and aquaculture production estimated from countries worldwide, some of which have diverse capture fishery and aquaculture activities. There are many small-holder fishers and farmers who contribute to the production making data collection difficult. That such a database would contain only approximate estimates of annual production should be intuitive. The reliability of the FAO agricultural database also has been questioned (Cafiero et al., 2014; Liu et al., 2020). While these databases have deficiencies, the user may not be able to identify discrepancies in the data, and there seldom is a way to correct suspected discrepancies. The two FAO databases are widely used by governmental agencies, non-governmental organizations (NGOs), scientific investigators, commercial enterprises, and other groups. These statistics are critical for assessing the current production, predicting future production, sourcing and planning, and environmental and resource conservation. While deficiencies exist within the FAO databases, these statistics are among the best available at present for global and regional assessments of food supply (ICC Library, 2021).

The estimates of meat yield from animals for human consumption used in this study were based on commercial processing data. Portions of the supply of different meats are purchased by consumers as whole animals or carcasses or reared on farms for family consumption or local sale, but we were unable to obtain a global estimate of the percentages. Home and on farm processing procedures are different from those in processing plants and may give different yields (Nelson, 2017; Ranches et al., 2020). It is general knowledge that people differ in eating habits, and this also will influence how much of the meat is actually consumed from a particular presentation of meat on their plates. In addition, an estimated 23% of meat production is lost and wasted. The majority (64%) of the loss and waste occurs at the consumer level, while 20% is in processing, 12% in distribution, and the rest is incurred at the farm level (Karwowska et al., 2021).

Thus, the data in Tables 1–5 are approximate. Nevertheless, they allow estimates of amounts of crude protein resulting from the several sectors of animal-source protein production to be compared.

### Comparisons

The present study revealed that aquaculture and capture fisheries combined to produce 15.3% of the global animal-source production for human consumption in 2018. The FAO (2020c) calculated that 17% of animal source protein was from aquaculture. The difference in the two estimates is not large considering that in both studies the percentage yields of meats for human consumption from whole animals or animal carcasses and of average protein concentrations of the meats had to be chosen from studies of which most allowed a range of percentages for both variables. We intentionally did not attempt to contact the individuals in FAO who made that estimate. By chance alone could the same estimates of meat yields and protein concentrations in meats have been selected in the two studies. By having two independent estimates which agree well, there can be more confidence of the contribution of capture fisheries and aquaculture to the global supply of animal-source protein.

Our results reveal that aquaculture does not lag much behind capture fisheries in protein production (Tables 4 and 5). However, capture fisheries are overfished and their production is not increasing, but aquaculture has experienced much growth since the 1950s (Fig. 1), and it is expected to continue to grow for the foreseeable future (Boyd & McNevin, 2015; FAO, 2020c).

The order of importance of protein production for human diets in both capture fisheries and aquaculture was finfish > crustaceans > molluscs (Figs. 2 and 3). Finfish provided 91.5% of the crude protein from capture fisheries, and freshwater fish were responsible for only 19.4% of finfish protein. The finfish category in aquaculture was separated into the species groups, and the carp species resulted in 58.4% of the farmed finfish protein. Crustaceans yielded about four times as much protein as did molluscs in capture fisheries, but only 2.5 times as much in aquaculture.

Aquatic animals are important in international trade, because some species are popular with consumers both for eating at home or in restaurants (Boyd & McNevin, 2015). Freshwater finfish and small marine finfish species from the capture fishery, and carps, tilapia, catfishes, and milkfish are particularly important as protein sources for lower-income families in developing countries (FAO, 2020c). From a food security perspective, fisheries and aquaculture production is most important in developing countries.

### Crude protein and protein quality

The comparison of quantities of protein from different foods or animal feeds using crude protein is problematic. Total nitrogen analysis measures both non-protein and protein nitrogen, but crude protein is estimated by the factor 6.25 (average ratio of protein to nitrogen in actual proteins) that is multiplied by total nitrogen concentration. In all except pure protein foods, crude protein overestimates true protein, and the argument against using crude protein as the standard for comparing protein concentrations among foods and feeds dates back at least to Forbes (1924) who suggested that the amino acid concentrations in a food or animal feed could be measured and summed to provide the true protein concentration. The same argument is still going on today (Hayes, 2020; Maehre et al., 2018), but Hayes (2020) states that crude protein is still the standard for comparison.

Alternate factors based on the ratios of the sum of amino acids to total nitrogen concentrations in different foods have been suggested as replacements for the crude protein factor 6.25 (Maehre et al., 2018; Ariňo et al., 2013). Mariotti et al. (2008) reviewed this topic and concluded the factors for different, major animal-source proteins should be as follows: chicken meat, 5.53; cattle meat, 5.48; fish, 5.48; milk, 5.72; eggs, 5.68; other sources, 5.60.

The sums from the amounts of protein from the major animal-source categories adjusted for the alternate factors of Mariotti et al. (2008) are given in Table 6. The estimate of total animal-source protein adjusted for nonprotein nitrogen is 10.3% less than that of crude protein. The adjusted protein amounts (Table 6) were 6.8% to 13.6% less than crude protein amounts. Crude protein gave the closest estimate of actual protein in milk. It gave a closer estimate of actual protein in eggs and aquatic animal meats than it did for terrestrial animal meats (Table 6). Among the meat animals, specific alternate factors for converting total nitrogen to protein were found only for pig and chicken meat, and only the factor 5.48 for fish and the factor 5.6 for other meats (Mariotti, 2008). Considering the information available and the amounts by which crude protein over-estimated actual protein, we believe that the differences shown in Table 6 do not invalidate crude protein for comparing the contributions of different sources to the global protein supply. This conclusion is supported by the statement by Hayes (2020) that crude protein is still the standard for estimating crude protein concentrations in foods.

**Table 6:** Comparison of crude protein amounts (kilotonnes) with protein amounts adjusted for nonprotein nitrogen

Protein source	Crude protein (total nitrogen × 6.25)	Protein (total nitrogen × alternate factor)	(% less)
Terrestrial meats	37,391	32,917	13.6
Eggs	8,686	7,894	10.0
Milk	30,889	28,912	6.8
Capture fisheries	7,135	6,518	9.5
Aquaculture	6,815	6,214	9.7
Total	90,916	82,455	10.3

^1^Chicken meat, 5.53; cattle meat, 5.48; fish, 5.72; eggs, 5.68; milk, 5.85; other protein sources, 5.60 (Mariotti et al., 2008)

A potentially greater concern when comparing protein sources is the quality of the proteins being compared. Protein quality is associated with digestibility of proteins and their amino acid balance with respect to human daily amino acid requirements (FAO, 2011; Schaafsma, 2000). The digestibility of major proteins are: terrestrial meats, 80.1 to 97.0 (Faber et al., 2010; Mendes et al., 2016); eggs, 90.9% (Evenepoel et al., 1998); milk, 95.0% (Dupont & Tome, 2014); fish, 95.1% (Deng et al., 2016); shrimp, 93.7% (Dayal et al., 2013); molluscs, 79.2% (Wang et al., 2019). Other than for molluscs, aquatic animal proteins are similar to terrestrial meat, eggs, and milk in digestibility. The protein digestibility-corrected amino acid score (PACAAS) is widely used for assessing protein quality (Schaafsma, 2000). Animal-source proteins typically have higher PACAAS scores than do plant proteins (Berrazaga et al., 2019; Herreman et al., 2020; van Vliet et al., 2015). Nevertheless, not enough information was found to allow a comparison of PACAAS scores among the different protein sources in the present study.

**Fig. 5:**
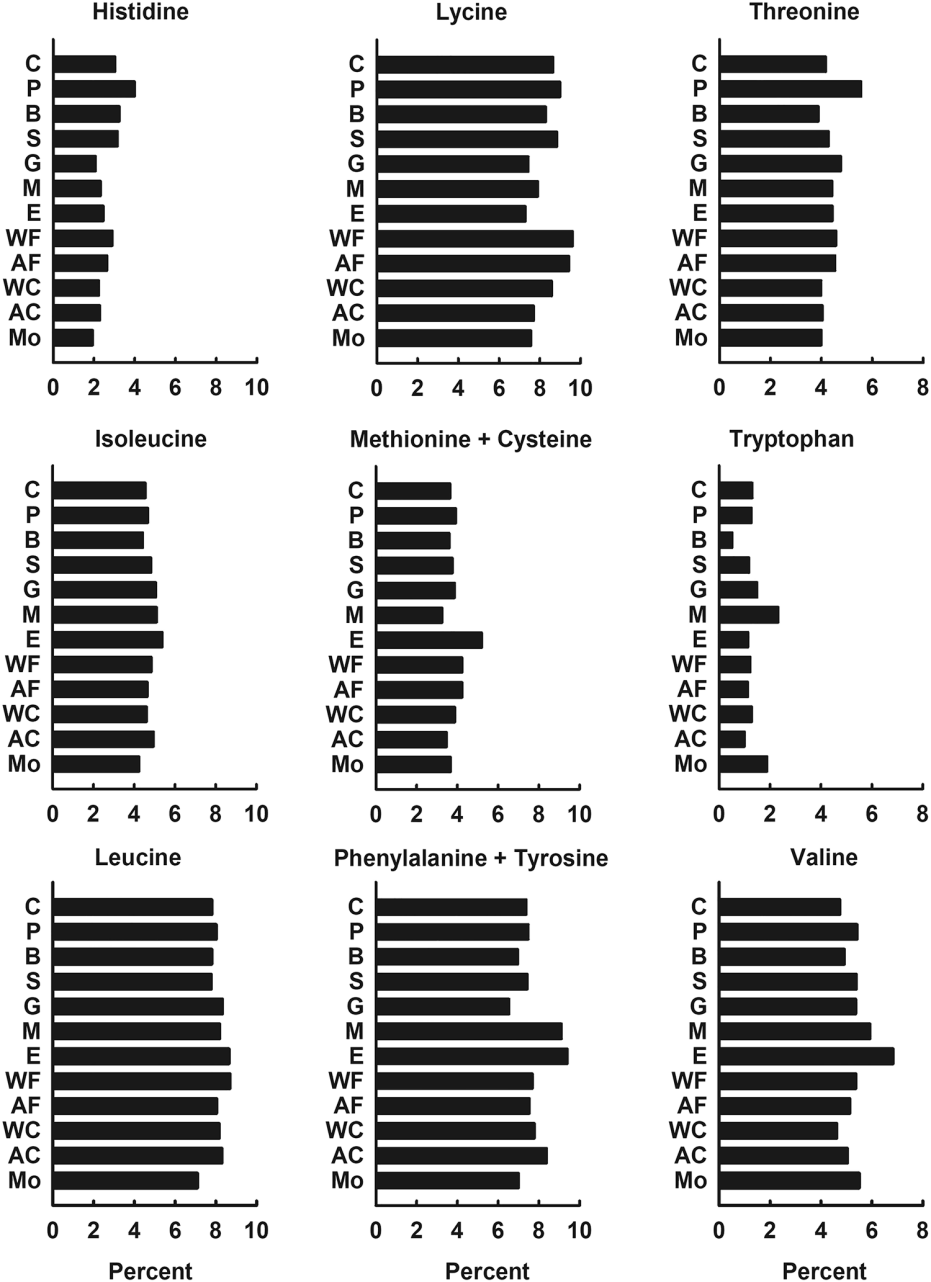
Average essential amino acid concentrations as percentages of protein for major sources of terrestrial and aquatic meat protein (source: USDA, 2021). Legend: C = chicken; P = pig; B = beef cattle; S = sheep; G = goats; M = milk; E = eggs; WF = wild (captured fish); AF = aquacultured fish; WC = crustaceans from capture fisheries; AC = aquacultured crustaceans; MO = molluscs from capture fisheries and aquaculture

While essential amino acid concentrations of different meats, eggs, and milk are generally similar (Fig. 5), there are some noticeable differences: pig meat is high in histidine and threonine; fish are high in lysine; eggs are particularly high in methionine plus cysteine as well as valine; milk and molluscs are high in tryptophan. Cattle meat is particularly low in tryptophan, and molluscs are low in leucine. The essential amino acid composition of wild-caught and farmed fish and crustaceans also is similar. Molluscs are especially high in tryptophan and low in leucine and phenylalanine plus tyrosine compared to fish and crustaceans.

**Fig. 6:**
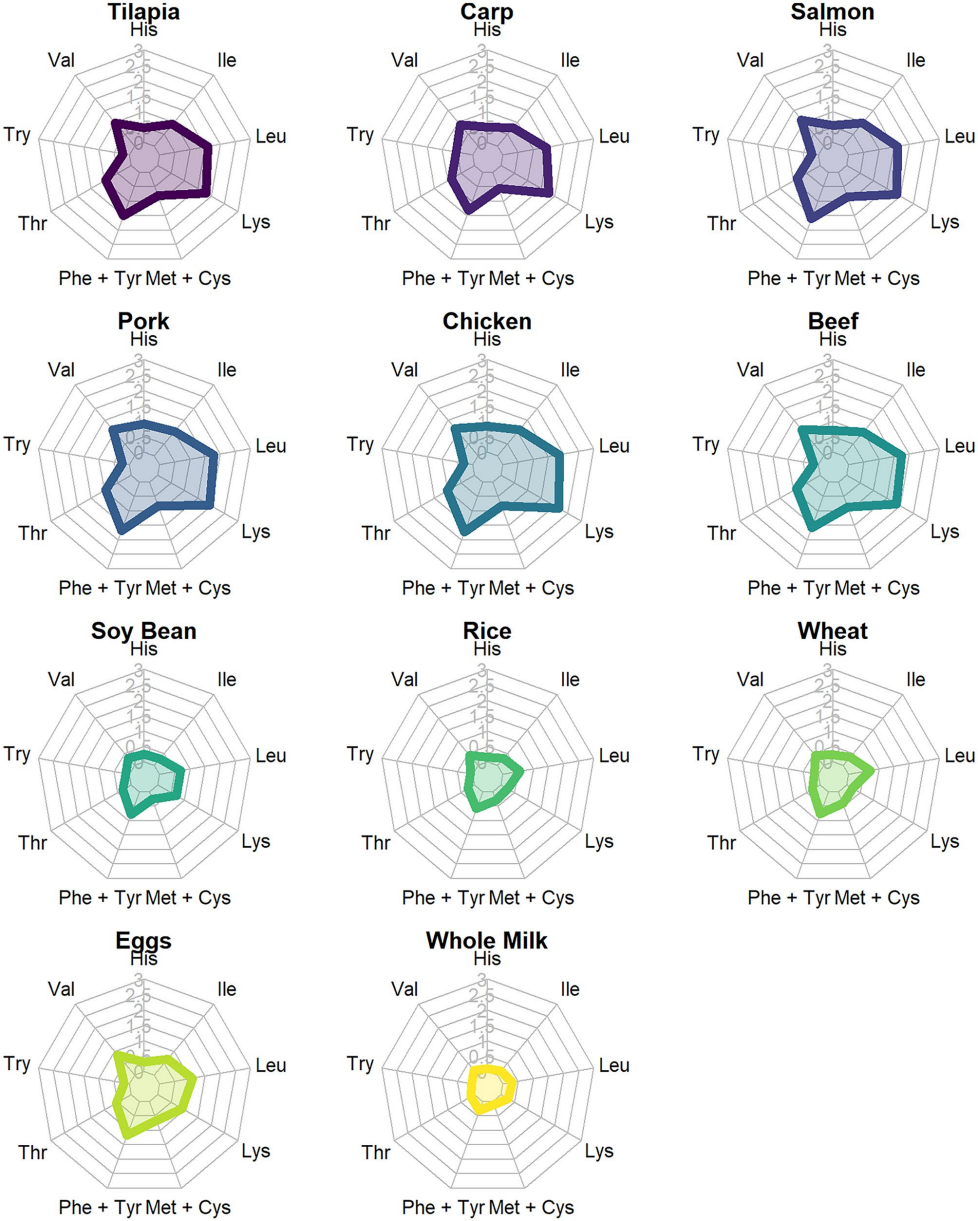
Spider plot comparisons of essential amino acid patterns in selected animal-source proteins. All values were obtained from the USDA’s FoodData Central database (USDA, 2021b) with the exception of carp, which were calculated from values for bighead carp in Pyz-Lukasik and Paszkiewicz (2018). All values are presented in g/100 g of tissue

Amino acid patterns in selected proteins were presented visually by aid of spider charts (Fig. 6). The lines extending outward from the centers of the spider charts show the essential amino acid concentrations, and the pattern produced in the central area of each chart by connecting the concentrations of amino acids depicts the amounts and pattern of the amino acids. There is much similarity among fish and terrestrial meats in the size and shape of the patterns. The shapes of the patterns also are similar among meats, milk, and eggs.

**Fig. 7:**
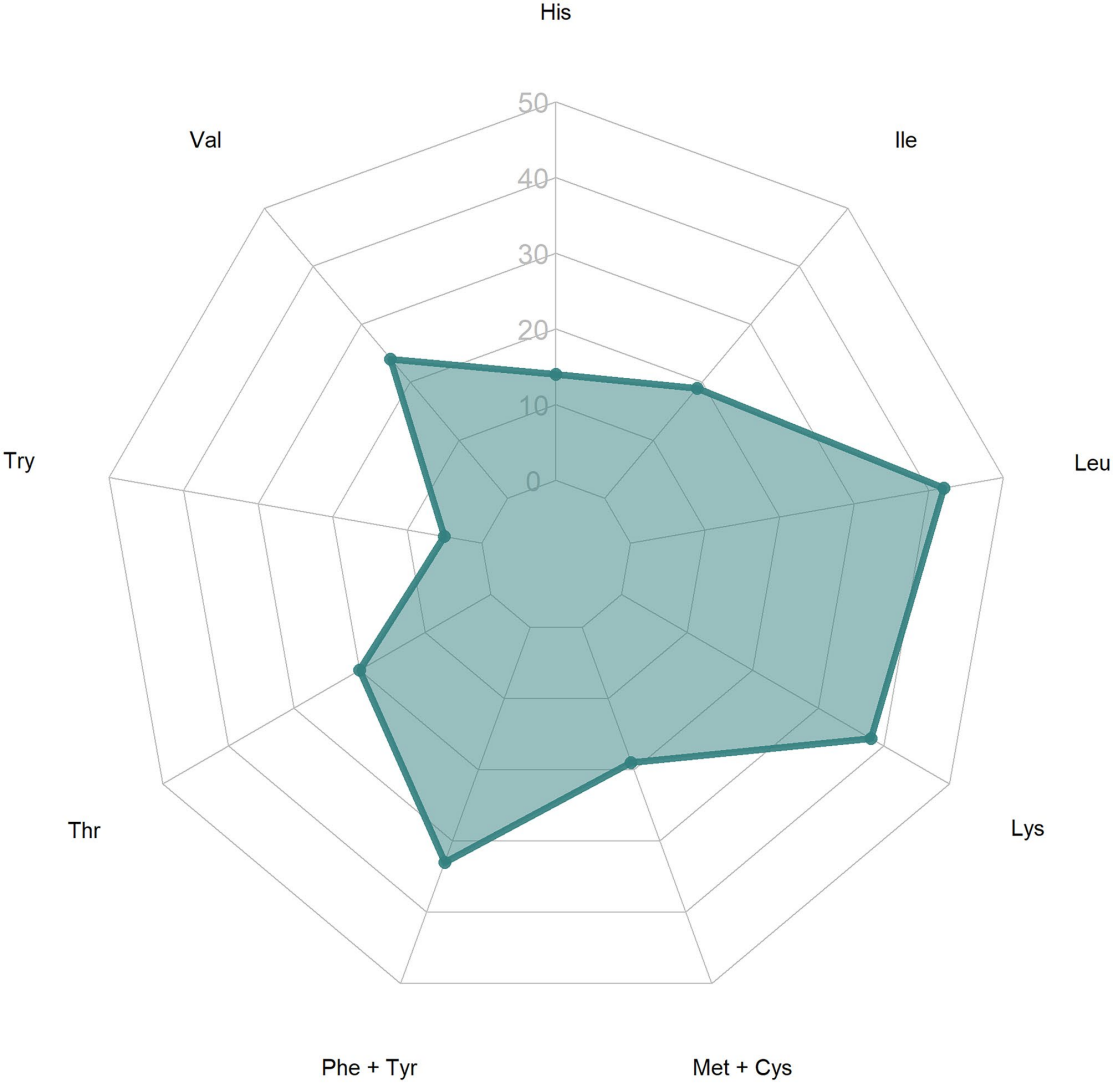
Spider diagram of the daily essential amino acid requirements in human diets, in mg of amino acid/kg of body weight, from Lupton et al. (2002)

The patterns for plant proteins are smaller and shaped differently from those of animal proteins. The sizes and shapes of the patterns of the animal proteins resemble those obtained using the average daily amino acid requirements for humans (Lupton et al., 2002) shown in Fig. 7 more than do those of plant proteins. This is visual evidence of the reason animal proteins are considered important in human diets.

Proteins from captured and farmed finfish and shrimp are of similar quality to those of terrestrial animal proteins. Thus, the direct comparison of the crude protein amounts resulting from the different animal categories seems reasonable, even when general protein quality is taken into account, as a way of comparing the relative contributions of the different types of animal meats to the global protein supply.

Aquaculture has been criticized for producing finfish with low concentrations of omega-3 fatty acids thought to protect against cardiovascular disease in humans (Alasalvar et al., 2002; Lenas & Nathanailides, 2011). This phenomenon likely is a result of high inclusion rates of plant ingredients in feeds and it can be offset by improved diet formulations (Miller et al., 2008; Santigosa et al., 2020).

### Wild fish, fishmeal, fish oil and feeds

The landings of many species of small, pelagic oceanic fish are used mainly in fishmeal and fish oil production, much of which is destined to be ingredients in fish and crustacean feeds (FAO, 2020c). Naylor et al. (2021) assessed wild fish use in aquaculture production for 2017. The total use was 12,566 Kt wild fish or about 78.7% of the estimated amount of whole fish reduced to fishmeal and oil (FAO, 2020c). The fish in-fish out (FIFO) ratio is the ratio of aquaculture biomass produced divided by the quantity of wild fish necessary to make fishmeal and fish oil included in feed. The use of fishmeal and fish oil rendered from waste is not included in the FIFO calculation, because these products are considered to be recycled from the capture fisheries not used for human consumption (Boyd & McNevin, 2015). Naylor et al. (2021) gave the FIFO for feed-based aquaculture as 0.28 indicating that 0.28 t of wild fish were needed to produce 1 t of farmed fish and crustaceans with feeds. However, to get this number, they divided total wild fish used by the total production of the species groups reared by feed-based culture. A portion of the production of these species groups was not feed-based. If instead, the wild fish used is based only on the total amount of production resulting from use of feed, FIFO is 0.39. For all of aquaculture production of 2017 (FAO, 2020c is the reference point), FIFO is 0.16. Regardless of which way the FIFO is calculated, aquaculture has a significant dependency upon capture fisheries.

The overall FIFO for feed-based aquaculture was well below 1.0, but the FIFO for salmon, marine fish, trout, and eels exceeded 1.0 in 2017 (Naylor et al., 2021). These four groups accounted for 65.7% of wild fish use in feeds. Although shrimp had a FIFO of 0.82, shrimp feed included 24.1% of the wild fish used. Two species popular with consumers in the developed world, salmon and shrimp required 56.1% of wild fish, but only provided 22.7% of the feed-based production.

The average fishmeal and fish oil inclusion rates in feed for feed-based aquaculture were calculated from data presented by Naylor et al. (2021) as 5.8% and 1.7%, respectively. The average FCR for all feed-based production was 1.59. Since 1997, fishmeal and fish oil inclusion rates have declined markedly and FCR has improved (Naylor et al., 2021). As an example, in 1997, salmon and trout feed contained 43% fishmeal and 25% fish oil, while the corresponding values were 12% and 10% in 2017. The FCR of these two groups also improved from 1.4 to 1.3 (Naylor et al., 2021). Nevertheless, the amount of fishmeal and fish oil used in aquaculture feed increased between 1997 and 2017, because the greater production in 2017 required about 5.9 times more feed than in 1997.

Naylor et al. (2021) reported feed-based production of 32,297 Kt of finfish and crustaceans. The production required about 2,975 Kt fishmeal of which 1,419 Kt were from waste rendering. We calculated that because 65.9% of rendered fishmeal is from processing fish from the oceanic capture fisheries (IFFO, 2020), a minimum of 935 Kt of rendered fishmeal used originated as a by-product of capture fisheries, and this fraction contained 472 Kt crude protein. This amount of crude protein could be added to the amount for human consumption making the ocean capture clearly a greater source of crude protein than is aquaculture.

**Fig. 8:**
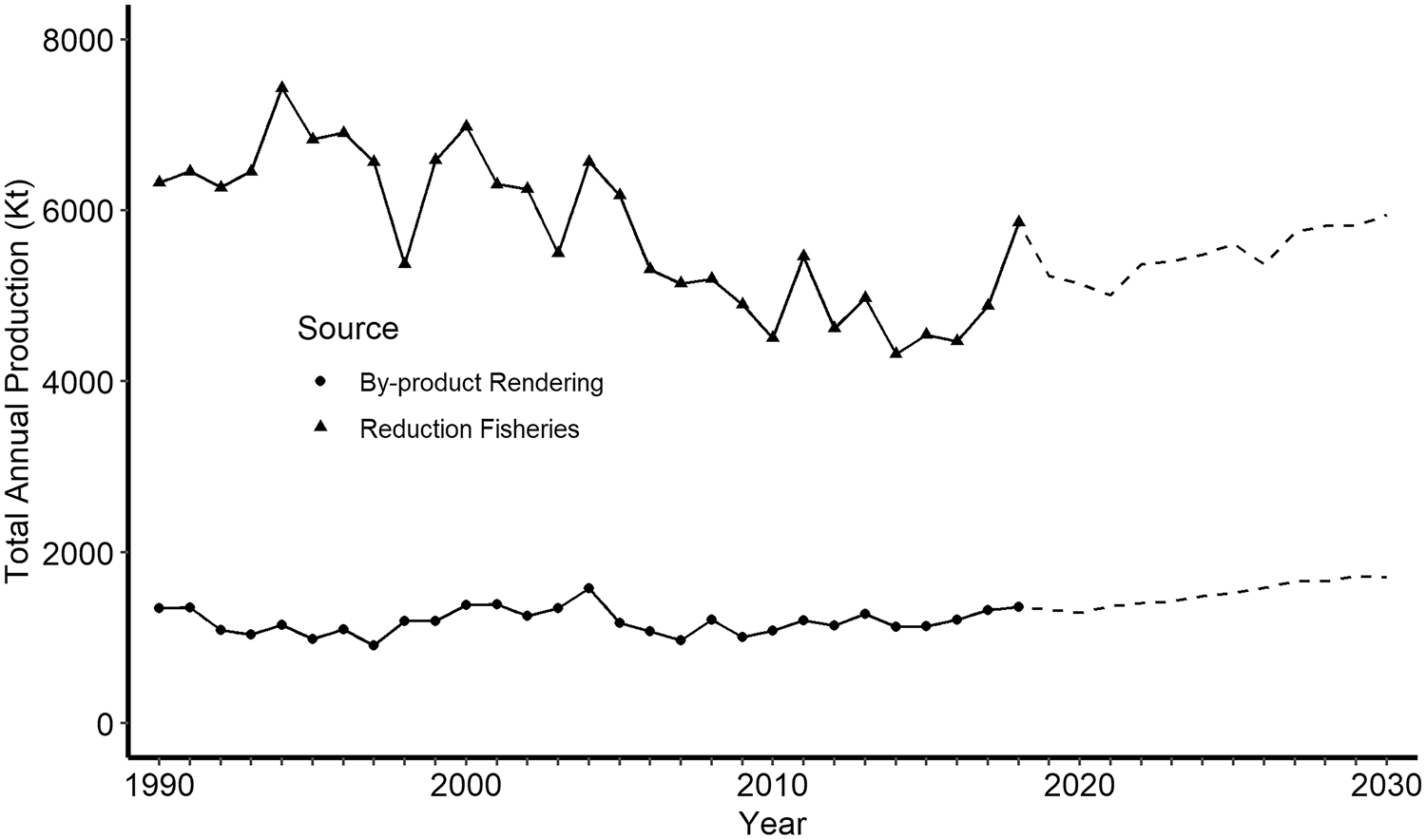
Global fishmeal production from 1990–2018 by source (circle = rendered from fish processing waste, triangle = from reduction fisheries). Projection of future production until 2030 is indicated by dashing of lines (source: FAO, 2020c)

The projected fishmeal production for 2030 is 6,000 Kt (Fig. 8) and based on the 2018 fish oil/fishmeal ratio of 0.235 calculated from data in EUMOFA (2021), there would be about 1,410 Kt of fish oil. The 1,800 kg of fishmeal from rendered by-processing waste has the equivalent crude protein content of 1,385 Kt of crude protein when compared to fishmeal made from whole fish. This amount of fishmeal would allow the 2017 production of feed-based aquaculture at an inclusion rate of 2.6% in feed—roughly half the 2017 inclusion rate reported by Naylor et al. (2021). It also would be enough fish oil at an inclusion rate of 0.69% in feed which is also about half of the 2017 inclusion rate. The potential for rendering processing waste is unrealized, because according to Jackson and Newton (2016) only 32.5% of these wastes are utilized for fishmeal and fish oil production. However, the utilization rate was reported by Naylor et al. (2021) to be increasing. These observations suggest that it might be possible to eventually wean aquaculture from whole wild fish completely.

The use of large amounts of wild fish for aquaculture feed ingredients has environmental and food security implications. Pauly et al. (1998) explained that the capture of larger fish from the ocean has declined in response to fishing pressure, and smaller fish from lower trophic levels were comprising increasingly more of oceanic landing. They referred to this phenomenon as “fishing down marine food webs.” The negative effects of the reduction fishery continue to be of concern (Pauly, 2012; Pikitch et al., 2014; Shannon & Waller, 2021; Cashion et al., 2017). Concern also exists because most of the wild fish used for fishmeal and oil production are suitable for human consumption (Alder et al., 2008; Cashion et al., 2017). Soliman et al. (2017) also argue that removing small fish from the ocean for fishmeal and fish oil production exacerbates the effects of climate change.

While feed-based aquaculture is an important component of world animal-source protein production, supplying wild fish to make feed ingredients to support its production has become problematic in marine conservation and food security. Moreover, the growth of feed-based aquaculture has a future limit that will be imposed by a shortage of wild fish for use in feed unless progress continues in reducing wild fish use.

### Seaweeds and molluscs

The harvest of seaweed from the ocean is mainly from aquaculture (Table 3). Although about 80% of seaweed production is considered for food use, at least half of this portion is raw material for extraction of hydrocolloids which do not contain protein but are used in human food and cattle feeds (Ferdouse et al., 2018; West et al., 2016). Seaweed culture does not require feed, fertilizers, and pesticides, and they absorb nitrogen and phosphorus (the nutrients responsible for eutrophication), and carbon dioxide which is a GHG. The carbon dioxide concentration in the ocean is rising because of increasing atmospheric carbon dioxide concentration and causing acidification (Doney et al., 2020). Duarte et al. (2017) has found that the parts of seaweeds that break off during growth are broken down into smaller pieces, and a portion settles into the deep ocean. High pressure, low temperature, and low dissolved oxygen concentration in the ocean depths are unfavorable to rapid microbial decomposition. As a result, seaweeds play a role in carbon sequestration in the deep ocean.

Molluscan aquaculture also does not require feed or fertilizer applications, and molluscs effect a net reduction in particulate nitrogen and phosphorus through filtration of suspended organic particles from water (Shumway et al., 2003). Xiao et al. (2017) reported that seaweed culture in the coastal waters of China removed 75 Kt/yr of nitrogen and 9.5 Kt of phosphorus.

Verdegem (2013) determined that seaweed and molluscan aquaculture, contrary to finfish and crustacean aquaculture, resulted in a net removal of nitrogen and phosphorus from natural waters. Farming of molluscs and seaweed in the ocean should be encouraged, but the concentration of protein is much lower in seaweeds, and molluscs have a slightly lower protein concentration and a much lower meat yield than do finfish and crustaceans. Thus, a several-fold greater production of molluscs would not provide a large amount of animal-source protein. Of course, on a local basis, there are many coastal bays where more molluscan culture would benefit community food security and livelihoods (Oliver et al., 2013). Likewise, a large increase in seaweed production would not yield a major quantity of plant-source protein.

### Intensification of aquaculture production systems

Aquaculture production increased rapidly from 1970 to 1990 and even more rapidly since 1990 (Fig. 1). The increase has been highly dependent upon feed-based aquaculture, and had it not occurred, the greater demand for seafood by a growing and more affluent global population could not have been satisfied (Boyd & McNevin, 2015). The increase in future demand can be satisfied through intensification of aquaculture production systems, expansion of the production area, or both. Expansion of the production area would increase land and water use (Boyd & McNevin, 2015; Verdegem & Bosma, 2009). This suggests that intensification of production systems may be a better choice, especially for pond aquaculture that is the major way of producing freshwater finfish and both freshwater and marine crustaceans at present (Boyd & Davis, 2020; Davis & Boyd, 2021a, b).

There is evidence from feed-based, coastal shrimp farming that intensification of pond production lessened the use of land and water (this includes agricultural land and freshwater necessary for feed ingredients) per tonne of production (Boyd et al., 2017, 2018b, 2021). In ponds where feed was applied and mechanical aeration also was used, increasing the level of intensification did not result in more energy use per tonne of shrimp in most feed-based culture, because the production increase possible per horsepower of aeration remains constant (Boyd & McNevin, 2020). Nevertheless, feeding and aeration result in a large energy expenditure because of electricity or diesel fuel use by aerators which must be supplied at 1 hp per 400–500 kg of standing biomass of shrimp and most species of finfish (Boyd & McNevin, 2020; Boyd et al., 2018a, b). In addition, feeds contain a large amount of embodied energy in comparison to other animal feeds (Chatvijitkul et al., 2017a). Finfish aquaculture in feed-based ponds is done by the same methodology used in feed-based shrimp culture (Boyd & Tucker, 1998), and the findings from shrimp aquaculture are applicable.

The previous paragraph provides a dilemma. Is it better to use more land and freshwater by expansion of the current production area to allow lower production intensity in the future, or is the better choice to use more energy to increase production intensity and conserve land and freshwater in the effort to meet future demands? We do not know the answer to this question, but believe that intensification would result in a better resource use tradeoff. Boyd and McNevin (2015) pointed out that aquaculture ponds often are located in areas of higher biological diversity than are croplands to produce feed ingredients, and land is a valuable resource in limited supply. However, from the standpoint of GHG production, there is insufficient information on the contribution to GHGs by aquaculture to include this factor in arriving at our opinion.

The situation with future increase in aquaculture production is confounded by the fact that the capture fishery is overfished (Pauly, 2012; FAO, 2020c) and not increasing in production (Fig. 1). De Silva (2016) proposed that culture-based fisheries in which aquaculture hatcheries could produce small fish for stocking into streams and lakes in Asia could increase the inland capture fishery. While this method might become important in local situations to increase inland capture fisheries, we believe that the future increase in demand for aquatic meat animals will be met by normal aquaculture methods. The world human population is expected to be about 30% greater in 2050 than in 2018 (United Nations, 2019). Assuming the *status quo* for per capita consumption, production for aquaculture and capture fisheries will need to increase by 46,915 Kt, and aquaculture production will need to increase from the 2018 production level by 57.2% to 129,000 Kt.

Aquaculture appears to be in the same situation today as agriculture was in the 1950s. A large population increase and need for more food loomed. As discussed by Boyd and McNevin (2015), agriculture rose to the challenge through intensification that required more fertilizers, animal feed, agrochemicals, energy, freshwater, etc. The amount of land used in agriculture today is only about 10% more than used in 1960, but food production has increased by 300%. Without this increase in land productivity, there would not have been enough food to support the growing population, but the process of intensification also required much greater resource use and caused tremendous environmental damage.

An assessment of aquatic foods with low negative impacts (called blue foods) by Gephart et al. (2021) identified farmed, bivalve molluscs and seaweed as the most environmentally benign sources. They also found silver and bighead carps to have the lowest GHG potential, but production of these finfish required considerable land and water. While Gephart et al. (2021) acknowledged that environmental trade-offs were important in assessing resource use and negative environmental impacts, they focused on low GHG emissions as the key indicator of blue foods. Seaweed and molluscs are but a minor source of protein even though they represent a large amount of the total production by capture fisheries and aquaculture. Doubling or tripling their production would not allow a large reduction in the amount of finfish and crustacean production in the future. Moreover, carps are not a widely sought food by consumers in more affluent countries (Boyd & McNevin, 2015; FAO, 2020c).

The assessment by Gephart et al. (2021) can be useful in efforts to inform consumers of the relative environmental performance of aquatic foods and to encourage wise environmental choices. The study also revealed the great need for a rational method of weighting land, water, energy, and wild fish use as to their importance in environmental sustainability.

The future demand for more food could be lessened or even negated by conservation measures and changes in eating habits. Gustavsson et al. (2011) reported that about one-third of world food production is wasted. Moreover, according to Richie and Roser (2017) many people eat more than required nutritionally, and among the global, adult population, 39% are overweight and another 13% are obese. Plant protein isolates are increasingly used in foods, and continued growth in the demand for these products would lessen the future demand for animal-source protein (Gorissen et al., 2018; Henchion et al., 2017).

The world food system has produced more than enough food to meet the global demand on a *per capita* basis during the past few decades (Boyd & McNevin, 2015). However, around 10% of the world population is undernourished because of poverty, conflicts in countries with weak governments resulting in inadequate distribution, and weather-related events (Action Against Hunger, 2021; Steenbergen et al., 2019). In coastal areas of developing countries many of the poor rely on small fish as a source of protein (Alder et al., 2008). Feed-base aquaculture, irrespective of its importance in the global protein supply, is thought also to be a negative factor in food security because of its dependency on wild fish.

While much emphasis has been put on lessening the use of fishmeal and fish oil from the reduction fishery in fish and shrimp feed, several species groups within feed-based aquaculture, carps, tilapia, and catfishes, use very little of this resource (Naylor et al., 2021). Production of species that require little or no wild fish are encouraged (Gephart et al., 2021; Naylor et al., 2021). Of course, current aquaculture feeds have high inclusion rates of plant meals, and fishmeal and fish oil often are replaced with soybean meal or other plant meals and vegetable oils (Davis, 2015). Thus, as with feed-based production of terrestrial animals, feed-based aquaculture competes with the use of plants for feeding humans (Schader et al., 2015).

### Pollution

Waste from feeding is the major source of water pollution caused by aquaculture (Tucker & Hargreaves, 2008). Chatvijitkul et al. (2017b) reported that each tonne of feed-based production resulted in 323–514 kg carbon, 35.9–63.5 kg nitrogen, and 6.1–15.9 kg phosphorus. The waste enters the culture system, and effluents from culture systems containing dissolved and particulate organic matter (source of biological oxygen demand or BOD), ammonia nitrogen, dissolved inorganic phosphate, and carbon dioxide can result in eutrophication (Boyd & Tucker, 1998; Cao et al., 2007; Dauda et al., 2019; Tucker & Hargreaves, 2008). Of course, some of the waste may be assimilated by natural processes within the production system, and a portion of the waste may be removed by sedimentation or filtration (Dauda et al., 2019; Tucker & Hargreaves, 2008). Only in cage culture do all the feeding waste discharge directly to the receiving water body (Verdegem, 2013).

Effluents from aquaculture can be a major source of pollution and contribute to eutrophication in areas with large amounts of production (Cao et al., 2007; Páez-Osuna et al., 1998; Tucker & Hargreaves, 2008; Verdegem, 2013). Because aquaculture often takes water in and discharges it back into the same source, reducing the water pollution potential of effluents from aquaculture farms can be beneficial to the environment and to the quality of water sources for aquaculture use (Boyd & McNevin, 2015). Improving the FCR to lessen feeding waste and selecting feeds that contain no more nitrogen and phosphorus than necessary, reduce the loads of these two nutrients in effluent (Chatvijitkul et al., 2018; Gross et al., 1998; Tucker & Hargreaves, 2008).

## Conclusion

Capture fisheries and aquaculture are important to the global animal-source protein supply, but only aquaculture is growing in its contribution to the supply. Finfish and crustaceans provide much more protein than do molluscs and seaweeds. About 75% of finfish and crustacean production by aquaculture is feed-based. While fishmeal and fish oil inclusion rates in aquaculture feeds and FCR values have improved over the past two decades (Naylor et al., 2021), because of greater production, aquaculture still is the major consumer of fishmeal and fish oil. The capture of wild fish to make fishmeal and oil results in perturbations in the marine food web and contributes to food security concerns as the wild fish could be used for human food. Ideally, fishmeal and fish oil use in aquaculture should be reduced to a level from which it no longer depends on the capture of small fish but relies only on fishmeal and fish oil from fish processing wastes.

Aquaculture production must increase nearly 60% above its 2018 production to supply the amount of seafood for the projected 2050 global population. This increase could be realized by intensification of production within existing facilities, expansion of the number or sizes of existing facilities, or both. The better of these three options is unclear. Ocean aquaculture of seaweeds and molluscs should be encouraged, because net removal of carbon dioxide, nitrogen, and phosphorus from the ocean by these two groups would offset some of the pollution caused by feed-based aquaculture. Seaweeds also effect carbon dioxide removal from the ocean, and leaves (fronds) which break off during growth lead to greater carbon sequestration in the deep waters of the ocean. By reducing wild fish use, encouraging seaweed and mollusc production, and better management methods to reduce water pollution, the sustainability of aquaculture could be improved. These measures also might allow some growth in capture fisheries.
